# α-Calcium Calmodulin Kinase II Modulates the Temporal Structure of Hippocampal Bursting Patterns

**DOI:** 10.1371/journal.pone.0031649

**Published:** 2012-02-20

**Authors:** Jeiwon Cho, Rushi Bhatt, Ype Elgersma, Alcino J. Silva

**Affiliations:** 1 Departments of Neurobiology, Psychiatry, and Psychology, Brain Research Institute, University of California Los Angeles, Los Angeles, California, United States of America; 2 Center for Neural Science, Korea Institute of Science and Technology, Seongbuk-gu, Seoul, Korea; 3 Department of Neuroscience, University of Science and Technology, Seoul, Korea; 4 Yahoo! SDC, Embassy Golf Links Business Park, Bangalore, India; 5 Department of Neuroscience, Erasmus MC, Rotterdam, The Netherlands; Georgia Health Sciences University, United States of America

## Abstract

The alpha calcium calmodulin kinase II (α-CaMKII) is known to play a key role in CA1/CA3 synaptic plasticity, hippocampal place cell stability and spatial learning. Additionally, there is evidence from hippocampal electrophysiological slice studies that this kinase has a role in regulating ion channels that control neuronal excitability. Here, we report *in vivo* single unit studies, with α-CaMKII mutant mice, in which threonine 305 was replaced with an aspartate (α-CaMKII^T305D^ mutants), that indicate that this kinase modulates spike patterns in hippocampal pyramidal neurons. Previous studies showed that α-CaMKII^T305D^ mutants have abnormalities in both hippocampal LTP and hippocampal-dependent learning. We found that besides decreased place cell stability, which could be caused by their LTP impairments, the hippocampal CA1 spike patterns of α-CaMKII^T305D^ mutants were profoundly abnormal. Although overall firing rate, and overall burst frequency were not significantly altered in these mutants, inter-burst intervals, mean number of intra-burst spikes, ratio of intra-burst spikes to total spikes, and mean intra-burst intervals were significantly altered. In particular, the intra burst intervals of place cells in α-CaMKII^T305D^ mutants showed higher variability than controls. These results provide *in vivo* evidence that besides its well-known function in synaptic plasticity, α-CaMKII, and in particular its inhibitory phosphorylation at threonine 305, also have a role in shaping the temporal structure of hippocampal burst patterns. These results suggest that some of the molecular processes involved in acquiring information may also shape the patterns used to encode this information.

## Introduction

A number of studies indicate that autophosphorylation at threonines 305/306 regulate the association of α-CaMKII with the post-synaptic density (PSD) [1], [2]. Autophosphorylation at 305/306 inhibits the interaction between α-CaMKII and the PSD, and appears to provide a negative constraint for long-term potentiation or LTP [1]. Mice with a mutation in the α-CaMKII gene, which replaces threonine 305 with an aspartate (α-CaMKII^T305D^ mice) and mimics inhibitory autophosphorylation [1], reveal hippocampal LTP deficits and learning and memory abnormalities in hippocampal tasks. Interestingly, preventing inhibitory autophosphorylation by replacing the α-CaMKII threonines 305 and 306 with nonphosphorylatable amino acids decreased the threshold for LTP induction and also resulted in learning deficits [1]. These findings demonstrate the importance of α-CaMKII inhibitory phosphorylation for synaptic plasticity and learning.

Besides its role in synaptic plasticity, α-CaMKII is also thought to affect the function of channels that modulate neuronal excitability [3], [4], [5], [6]. Thus, it is possible that a mutation which prevents the activation of this kinase and disrupts its cellular distribution could also affect neuronal excitability and possibly the temporal structure of *in vivo* burst patterns, especially in the hippocampus, a region that expresses high levels of α-CaMKII [7]. In this paper, we describe experiments with *in vivo* extracellular recordings of awake and behaving mice that indicate that α-CaMKII has a role in bursting patterns of hippocampal pyramidal cells.

The hippocampus has a critical role in spatial learning and memory. Hippocampal cells show unique place-dependent firing characteristics that are thought to reflect spatial cognitive maps [8], [9], [10], [11], [12], [13], [14]. Place fields form rapidly as an animal navigates in a novel environment [15], and they tend to be stable over time [16], [17]. Pyramidal neurons in the CA1 area of the hippocampus fire single spikes and complex bursts of consecutive spikes as an animal navigates through a given environment [18], [19].

Several studies have demonstrated that the mutation of single genes required for synaptic plasticity disrupt the stability of hippocampal place fields [20], [21], [22], [23], [24], [25]. We extend these findings and provide evidence that α-CaMKII, a gene required for synaptic plasticity, place cell stability and learning and memory, is also involved in modulating place cell firing characteristics. Our results reveal that α-CaMKII is involved in governing the temporal patterns of complex spiking as well as the reliability of spiking with respect to the animal's position. Therefore, our results provide *in vivo* evidence that the α-CaMKII gene has a role in shaping pyramidal cell spiking patterns.

## Results

CA1 area pyramidal cells were recorded extracellularly using single unit recordings and only those cells showing a distinct spatial firing preference were included in the analyses (>0.2 Hz of mean firing rate & >0.5 of spatial selectivity at the same time). Twenty-eight place cells were recorded from 6 α-CaMKII^T305D^ mice, and 32 place cells were recorded from 6 wild type littermates. Recordings were made while the animals were moving freely inside a cylindrical chamber 30 cm in diameter and 35 cm in height (see Methods for details). Along with neuronal recordings, positional data were also gathered through the video-tracking system (Datawave. Inc). No difference was observed in gross behavior including running speed (mean±S.E.M.: 6.44±0.22 for T305D vs. 6.15±0.17 cm/sec for WT, p = 0.29) between T305D and WT. This suggests that differences in place cell characteristics between WT and T305D mice were not the result of differences in running speed or related behaviors (e.g., overall activity level).

### General Properties of Place Fields are Normal in T305D Mice

**Figure 1:**
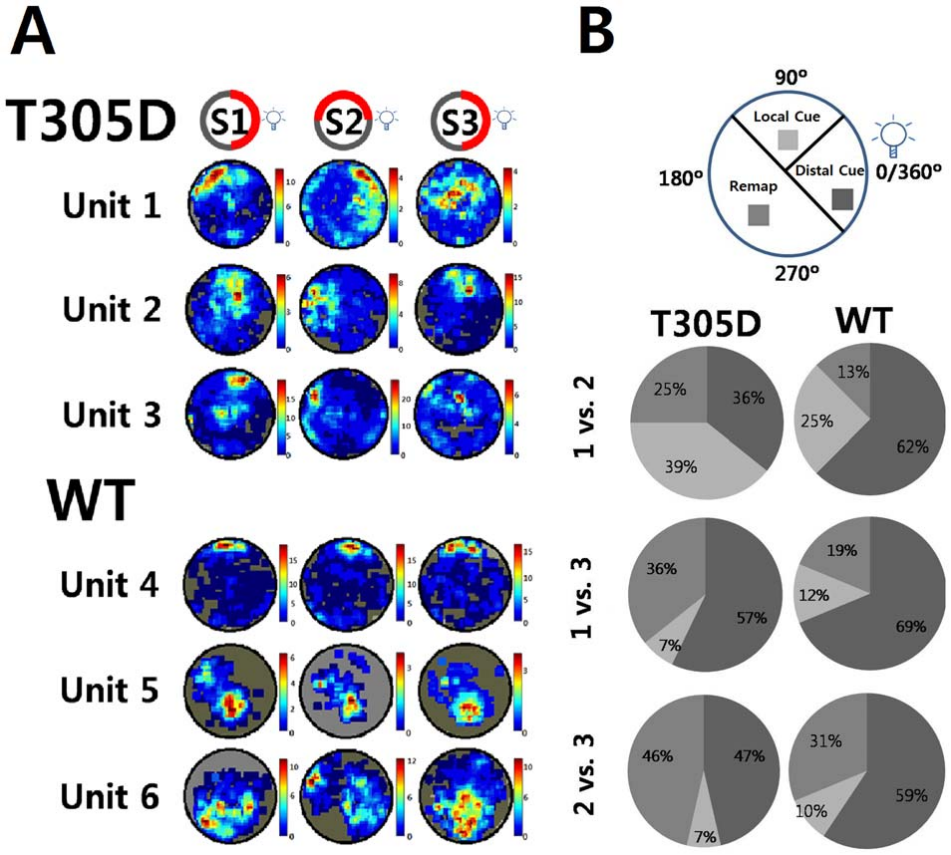
Comparisons of place fields. A. Examples of place fields from α-CaMKII T305D mice (top three rows) and their wild type counterparts (bottom three rows). Session 1 and 3 were identical in terms of visual cues available, while for session 2 a cue card in the wall of the arena was rotated by 90° in a counter clockwise direction. A distal cue (light) located outside the recording cylinder was never moved. S: Session. Firing Rate codes (Hz) were labeled on the right of each place map. B. Comparisons of place field stability between T305 mutants and controls. Place fields were classified according to whether they followed a Local Cue (card), Distal cue (light) or Remapped. The figure shows the proportion of place fields in each of these three categories (Local Cue, Distal Cue and Remapped). Local and distal cue configurations were as described above.

Figure 1A shows examples of place fields of hippocampal CA1 pyramidal neurons from both groups measured in 3 sessions. The configuration of visual cues was identical in sessions 1 and 3. In session 2 a cue card within the recording cylinder was rotated by 90° in a counter clockwise direction. However, distal cues outside of the recording cylinder were not moved. Our set up included a light positioned outside of the arena in the ceiling of the recording environment, which was a salient distal cue within the relative darkness of the recording environment.

Visually, the individual place fields from T305D mice appear to be normal; the complex spike neurons in the hippocampal CA1 of the mutants also displayed place-dependent increases in firing rates. Neuronal firing rates over whole sessions did not differ either (1.51±0.14 in T305D vs. 1.29±0.09 Hz in WT, p = 0.18). Although there was a slight tendency for increased firing rates in T305D mice, this did not reach statistical significance.

*In-field* firing rate of the place fields measured was defined as the average firing rate in position pixels within the firing field. The firing field pixels, in turn, were defined as those pixels for which the firing rate of the cell was higher than the average firing rate of the cell. The mean *in-field* firing rates did not differ significantly between mutants and WT littermates (3.66±0.25 Hz in T305 vs. 3.38±0.2 Hz in WT, p = 0.36). *Spatial selectivity* is the measure of the degree of the elevation of in-field firing rate as compared to firing outside of the field [Spatial Selectivity = *log*_10_ (InField Firing Rate/OutField Firing Rate)]. Average spatial selectivity of place fields was also not significantly different between T305D and WT mice throughout all sessions recorded (1.08±0.05 in T305D vs. 1.18±0.05 in WT, p = 0.18). When only burst spikes were used to calculate selectivity, we confirmed that there is no significant difference between groups (1.68±0.37 in T305D vs. 1.71±0.24 in WT, p = 0.77).

### Decreased Spatial Coherence and Information in Place Fields of T305D mice

Although the gross firing characteristics of cell spiking seemed normal and similar to WT, T305D mice showed on average significantly larger place fields than those in WT mice (231.77±10.31 vs. 161.82±6.66 pixels, p<0.00001, respectively). The place maps of T305D mice also showed higher pixel-to-pixel variability as measured by *spatial coherence*. Spatial coherence is a way to quantify the local smoothness of a place field surface. Spatial coherence of a place cell is defined as the correlation between firing rate of a position pixel and the average firing rate over its 8 neighboring pixels. For cells with high spatial coherence, firing rates of most position pixels are highly correlated with its neighbors, and consequently the cell-firing rate over a given spatial location is usually smooth. On the other hand, with a low spatial coherence, firing rates at nearby locations are uncorrelated and the firing rate map is highly variable from position to position. Spatial coherence was significantly lower in T305D mice than in WT littermates (Z transformed, 0.42±0.02 vs. 0.52±0.02, p<0.005, respectively). In addition, we measured spatial information [26] that provided us with information about animal's current position predicted by firing rate, as follows,
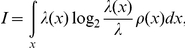
where *I* is the information rate (bits/sec) of the cell, *x* is location, *p(x)* is the probability density for the rat being at location x, 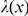 is the mean firing rate when the animal is at location *x*, and 
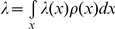
 is the overall mean firing rate of the cell.

Spatial information was also significantly lower in T305D mice than WT littermates (0.87±0.07 vs. 1.05±0.08 bit/sec, p<0.05, respectively) suggesting that place cells in T305D mutants process less information about the given environment compared to the ones in WT littermates.

### Decreased Stability of T305D Place Fields

Next, we asked whether place fields were stable in T305D mice. Using a pixel-by-pixel cross correlation across the 3 recording sessions (see Methods for details), stability or reproducibility of each place field was examined by comparing the average firing rates of each pixel across 2 place fields obtained from different recording sessions. The pixel-by-pixel cross-correlations of place fields across sessions reflect the stability of spatial representations. We used ∼5 min intervals between the end of sessions 1 and 2, and between the end of sessions 2 & 3. Approximately 35 min intervals separated sessions 1 & 3. Each session was ∼25 minutes long.

**Figure 2:**
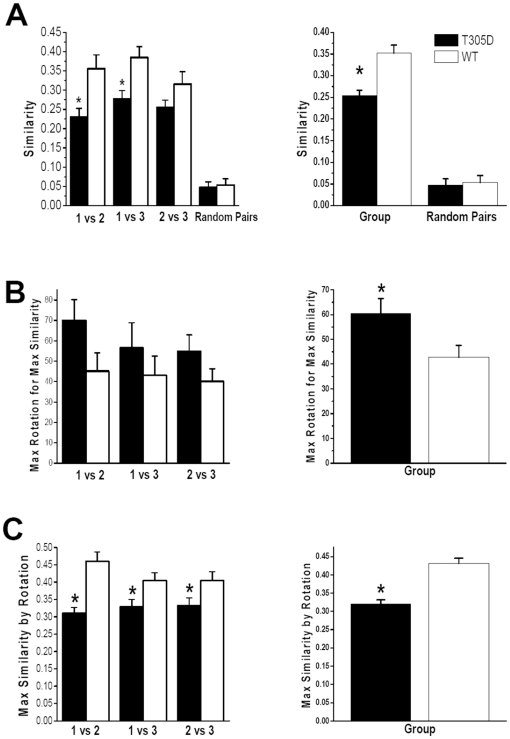
Comparisons of the likeness of place fields in T305 mutants and controls. Statistical measures to compare the likeness of place fields across sessions are shown on the left panel, and the same measures pooled across all sessions are presented on the right panel. A. Similarity reflects pixel-by-pixel correlation values for session pairs plotted on the horizontal axis in the left column. The same measure pooled across all sessions is shown on the right. B. Maximum rotation (in degrees) required for one field to be maximally similar to another. The rotation values are the absolute amount of rotation; e.g., counter clockwise as well as clockwise rotation of 90° have a value 90 in the plots. C. Similarity measure obtained after the aforementioned rotation operation. * indicates significant difference between groups at p<0.05.

T305D mice showed lower pixel-by-pixel similarity in place fields between sessions than their WT littermate controls (Figure 2A). Specifically, the similarity between session 1 and 3, which had identical cue card positions, was significantly lower in mutants than controls (p<0.05), suggesting that place cells in the mutants were less capable of recognizing the same environment compared to the control group. Similarities for other combinations (1 vs. 2 and 2 vs. 3) also showed differences between mutants and controls. The results indicate that WT place fields were far less affected by small environmental changes, (e.g., a cue rotation, interval between sessions) than T305D place fields. In another words, the place fields of mutant mice were more prone to remapping than those of controls. Nevertheless, we found that the similarity of place cells in the mutants was significantly higher than two randomly picked pairs of place cells, indicating that T305D place cells do not completely remap after the end of each session (compare Random Pairs similarities to the rest of bars in Figure 2A).

### Differences in Cue Dependency of Place Fields

To further study place field stability across recording sessions, we rotated place fields of one of the sessions being compared (session 1 vs. 2, 1 vs. 3 and 2 vs. 3) until we found the maximum similarity between the two sessions for the same cell. With a completely stable place field, we would expect that the maximum similarity would be obtained with zero rotation of one of the place fields. Cells with a higher amount of required rotation for maximizing similarity may be deemed less stable. Even though comparisons between individual sessions showed only a tendency for increased rotation required for T305D place fields compared to WT place fields (Figure 2B, left panel; p = 0.07 for 1 vs. 2, p = 0.38 for 1 vs. 3 and p = 0.15 for 2 vs. 3), the amounts of rotation required for maximum similarity were larger for T305D place cells. The difference between T305D and WT was more obvious when the comparison included all sessions (p<0.05), which is consistent with the earlier observation that place fields in T305D mice are less stable than controls (Figure 2B, right panel). These results were consistent over different measurements for similarity or stability of place fields. For example, the maximal amount of similarity achieved for T305D mice was significantly lower than that of WT (Figure 2C). These observations suggest that the 90° internal cue rotation in session 2 and/or the removal from the recording environment between sessions disrupted the place fields of the mutants more than WT littermates.

As a rough estimate of remapping, we also classified place cells according to the amount of rotation required for maximum similarity between recording sessions (Figure 1B). Our set up included a light positioned outside of the arena in the ceiling of the recording environment, which was a salient distal cue within the relative darkness of the recording environment. Place cells were divided into 3 categories for each comparison pair (1 *vs.* 2, 1 *vs.* 3 and 2 *vs.* 3). Following cue card rotation, place fields that stayed within a 90° range of their original position relative to a distal light cue outside of the recording cylinder were classified as a “Distal Cue” place fields. Place fields that moved within a 90° range of the new position of the cue card were categorized as “Local Cue” place fields. The rest of the place fields that did not stay in their original position or rotate with the cue card, were classified as “Remap” place fields. Our results (Figure 1B) show that many place fields in the WT group tended to stay in the same quadrant (followed distal cue) in spite of the local cue rotation (session 2), suggesting that for WT mice the prominent distal cue had a dominant role in shaping place field firing in the experimental setup used in the current study. Our results suggest that faced with the conflict between distal (did not change position between recording sessions) and local cues (changed between recording sessions), WT place cells tended to follow the prominent distal cue and stay in the original location, while T305 place cells did not. Instead, a larger percentage of T305D place cells either remapped or appeared to follow the local cue card. This result indicates that the T305 mutation affected the behavior of place cells in the mutants. It is possible that the conflict between the unchanging prominent distal cue and the changing local cue affected the place cells of the mutants more than the place cells of WT mice.

Altogether the data presented indicate that although modulation of firing rate was similar between groups (e.g., no change in spatial selectivity), T305D place fields were more variable (as measured by spatial information and coherence) and unstable.

### Abnormal Spiking Patterns in T305D Mutants

**Figure 3:**
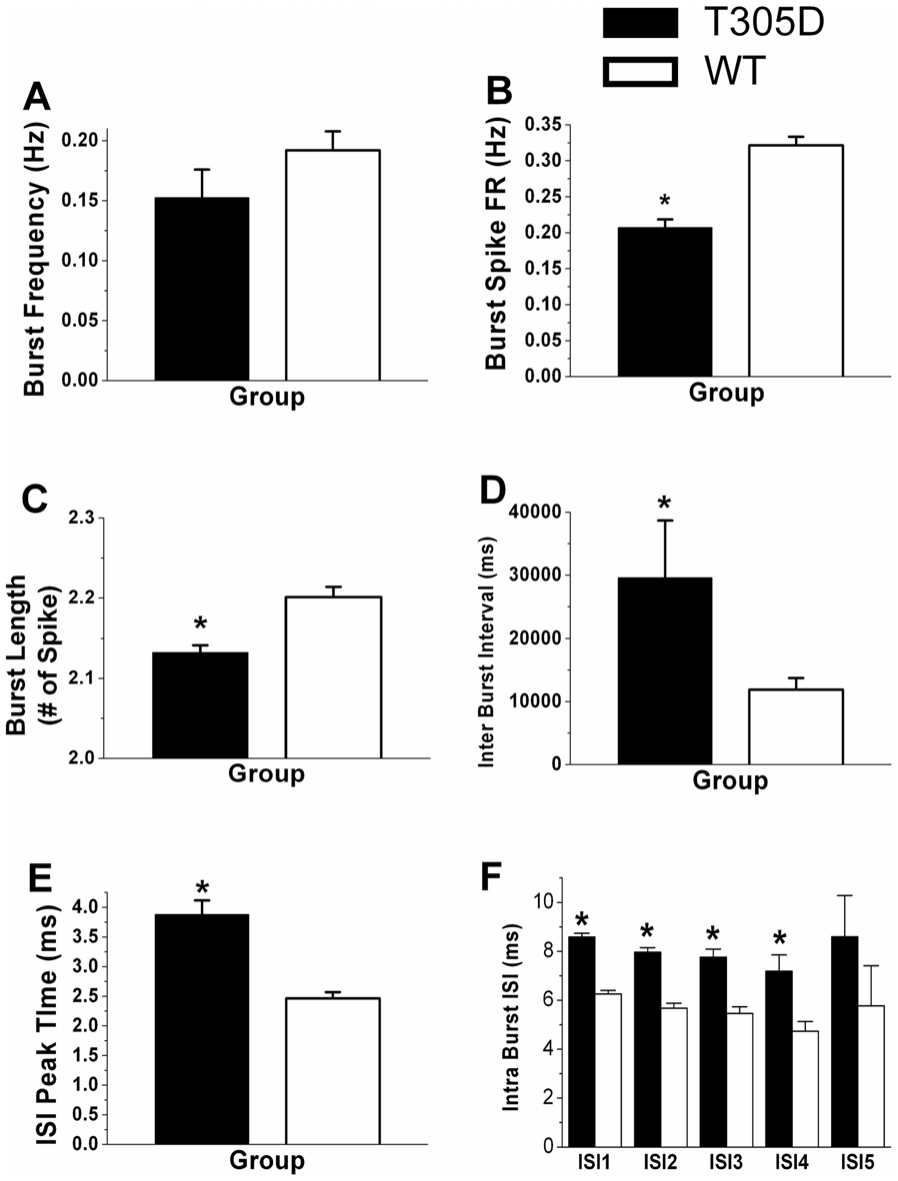
Comparisons of complex spikes between T305D and WT groups. A. Frequency of Burst (Hz) did not differ significantly, B. Firing rate of Burst Spike (Hz), the frequency of spikes within bursts was significantly less for T305D mice, C. Burst Length in terms of number of spikes per burst was significantly less for mutants, D. Average inter-burst intervals were significantly longer for T305D mice, E. Average peak time from individual ISI histograms are consistent with significantly prolonged intervals between overall spikes. F. Average Intra Burst Intervals show that intervals between two consecutive spikes in bursts were significantly prolonged in the mutants, * indicates significant difference between groups at p<0.05.

*In-vivo* extracellular recordings of CA1 pyramidal neurons in both T305D and WT mice showed a characteristic bursting pattern of two or more action potentials in quick succession with progressively diminishing amplitude. However, analyses of the peak time for inter-spike intervals (ISI) from individual neurons revealed that this value was higher in T305D mice than in controls (3.88±0.24 vs. 2.46±0.11 ms, respectively, p<0.0001, Figure 3E), suggesting that there are fundamental changes in the temporal spiking properties of pyramidal neurons of T305D mice.

#### Overall ISI characteristics

To better understand the characteristic differences in spiking between the two groups, we compared ISI histogram variability of all spikes in the recording session by obtaining the coefficients of variation (CV, the ratio of standard deviation to the mean of ISIs for measurement of the distributional dispersion) over the whole recording sessions. We found that over the whole session, the CVs of mutants and controls were not significantly different (2.31 for T305D vs. 2.23 for WT, p = 0.46). However, the entropy of the distribution increased significantly for T305D (10.68 bits for T305D vs. 9.7 for WT, p<0.01). Altogether these results indicate that even though the *overall* spiking distributions were not different between T305D mice and controls, the increased entropy suggests a higher amount of variation in ISIs of T305D mice.

We have also assessed the relation between the remapping phenomenon (Figure 1B) and the ISI peak time using a logistic regression analysis as follows. The Logistic is fit as log(P(Remap(i))/(1-P(Remap(i))) = a_0+a_1×ISI_Peak(i)+a_2×T(i)+a_3×Session2(i)+a_4×Session3(i), where a_0 through a_4 are regression coefficients. For neuron i, P(remap(i)) denotes the probability of the neuron undergoing a complete place field remapping between sessions as opposed to a less than 90deg rotation with either the local or distal cues. ISI_Peak(i) denotes the peak ISI time, T(i) = 1 denotes that i is in T305D, while T = 0 denotes that i is in WT group. Session2 and Session3 are indicator variables for sessions 2 and 3, respectively. The P(remap) when Session2 = 1 is thus the probability of a complete remap from session 2 to session 3. For this prediction, we used the ISI peak time for session 2. We found that a longer ISI peak time was a predictor for complete remapping (Coefficient = 0.2, p<0.05). In a reduced regression using Group and Session ID, we also confirmed that place cells in the T305D group tended to have higher probability of remapping (Coefficient = 0.61, p = 0.1). These results suggest that a prolonged ISI peak time may predict remapping and that remapping tends to occur more frequently for place cells in the T305D group.

#### Correlation of spiking rate given spatial position

Since we did not find any glaring differences in the ISI histograms over the whole recording session, we next asked how predictably place cells fired at each position pixel. We used spatial regression [27] to estimate firing rate at each position pixel. If spatial regression does a good job of predicting firing rate given the position at new places, one could say that the regression has captured the position-dependent firing rate characteristics of the place cell. A good regression fit would explain high amount of variance in firing rate across all position pixels.

We saw that the percent of variance in pixel-by-pixel firing rate explained by regression on position was lower for T305D mice compared to WT mice (Firing rate percent variance explained for T305D = 60.3% vs. WT = 67.7%, p<0.001). In other words, having access to the animal's position was less useful in predicting place cell firing rate for T305D mutants. This result is not very surprising, since spatial coherence for T305D mice was also lower. Spatial coherence measures the correlation in firing rate between a position pixel and the neighboring 8 pixels around it, over the whole area. The spatial regression utilized here differs from spatial coherence, since the data fit utilizes firing information over a larger number of pixels in the neighborhood of each pixel to predict its firing rate. The neighborhood size could potentially include the whole area, if necessary. We also adapt the neighborhood size for each cell, and fit using a neighborhood size through a smoothing parameter that yields the best results, as described in the next parameter. We think the spatial regression utilized here is a better estimate of inter-dependence of local firing rate of place cells than spatial coherence, since it is not dependent on pixel sizes but on the smoothness of the firing rate place field.

We now discuss the choice of spatial smoothing parameter. One of the shortcomings of spatial coherence or spatial information measures is that they are closely dependent on the size of each position pixel. Larger pixels would smooth the place field more, and result in higher spatial coherence or lower spatial information. Too small a pixel size on the other hand would result in lower spatial coherence or higher spatial information. Rather than comparing firing rate correlation between a pixel and its surrounding (i.e., spatial coherence) or departure in the distribution from uniform firing rate (i.e., spatial information), we asked what is the *best prediction job* one can do using available place dependent firing rate information *irrespective* of the extent of spatial neighborhood used for the prediction. With this thought, we think the simplest plausible method was to use a spatial regression with a smoothing parameter individually tuned to each place cell.

To arrive at the best smoothing parameter, we used a 10-fold cross-validation over a wide range of smoothing parameters and picked the best parameter for each cell. Generally, we found that small smoothing parameter values generally produced the best fit. Thus, the spatial correlation analysis also lends credence to the spatial coherence results reported earlier, which uses only the neighboring pixels for a linear regression fit. k-fold cross-validation is a standard technique in regression estimation [28], where data are split in *k* non-overlapping parts. Individual regression is fit *k* times while holding out one of the *k* parts, and prediction accuracy is measured on the held-out sample. The smoothing parameter that achieves the best average prediction on a held-out sample is then used for regression analysis.

Once the best smoothing parameter was found for each cell, we performed a regression analysis for each cell as follows: For each position pixel, the predicted firing rate was calculated as a *least-squares regression fit* with that smoothing parameter. The regression output was then compared with the actual firing rate at each position pixel, and the percent of firing rate variance was computed as follows:
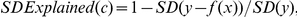
where 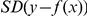 is the standard deviation of residuals of actual and regression fit of firing rates, and is the standard deviation of the firing rate over all pixels. As reported above, the percent variance explained in T305D mice was lower, suggesting that knowledge about firing rate in surrounding pixels was not as predictive for the mutants, even if we used a larger surrounding area for firing rate prediction.

#### Increased temporal variability of Intra Burst Spikes

To further understand the increased position-dependent firing rate unpredictability, we restricted our comparisons to spikes within bursts (Intra-burst spikes). Complex spike bursts are suggested to be the principal signal of positional information [29]. Therefore, we asked whether the temporal characteristics of spiking itself changed for T305D animals. Consistent with generally accepted definitions [16], any event of two or more spikes with each spike occurring within 15 ms of its predecessor with progressively decreasing amplitudes was termed a burst.

The frequency of bursting did not differ significantly between T305D and WT mice (0.15±0.02 vs. 0.19±0.02 Hz, p = 0.15, respectively, Figure 3A). However, the firing rate of spikes within bursts (intra burst spike rate over total recording time) in T305D mice was significantly reduced compared to that of WT littermates (0.21±0.01 vs. 0.32±0.01 Hz p<0.0001 respectively, Figure 3B), suggesting that the overall bursting activity of CA1 pyramidal neurons in T305D mice was disrupted. On average, bursts in T305D mice contained significantly fewer spikes compared to WT mice (2.16±0.003 vs. 2.22±0.003 #spike/burst, p<0.00001, respectively, Figure 3C). The ratio of intra-burst spikes to total spikes in each of the recording sessions, was also significantly lower in T305D mice than in WT mice (20.7±1% vs. 32.1±1%, p<0.00001, respectively) suggesting that single spikes are more frequent in the mutant mice. Inter-burst-intervals were also significantly longer in mutant mice than in wild type littermates (29.6±9.1 vs. 11.9±1.8 seconds, p<0.05, respectively, Figure 3D).

We also compared consecutive spike pairs in a burst: intra burst ISI-1 refers to the interval between the first and second spike, ISI-2 refers to the interval between the 2^nd^ and 3^rd^ spikes, etc. Intra burst spike intervals were significantly longer in the mutants (see Figure 3F).

**Figure 4:**
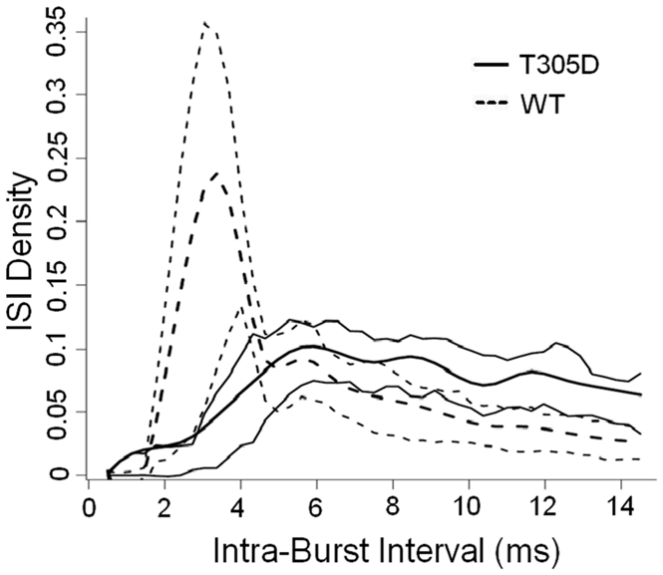
ISI density distribution of ISIs between the first and the second spikes of all bursts. Thick lines show the mean of individual ISI histograms of cells for the two groups, T305D (dashed line) and WT (continuous line). Thinner lines show 25^th^ and 75^th^ quartiles for cells in each group, over the range of observed ISIs. Intra burst ISI in T305D group is more variable compared to WT group.

Figure 4 shows smoothened mean intra-burst ISI histograms for T305D vs. WT mice. For clarity and to avoid redundancy, only ISIs between the 1^st^ and 2^nd^ spikes of a burst are shown, since we observed identical curves for ISI-2 and ISI-3. Smoothing was performed by fitting cubic splines to each cell ISI histogram, and then performing an average of these spline fits for each group to get the average for each group. Point-wise 25^th^ and 75^th^ percentile of these spline fits constitute the thin-lines. Thinner lines in Figure 4 show the 25^th^ and 75^th^ quartiles for each ISI value. We quantified this increased variability using Entropy. For each cell and session, we measured the entropy of the ISIs. For example, ISI-1 is the entropy of the distribution of ISIs between the 1^st^ and 2^nd^ spike of a burst. A two-tailed t-test comparison showed that the ISI-1 entropies of the T305D group were significantly higher than controls (3.9 bits for the T305D group vs. 3.5 bits for the WT mice, p<0.01). This shows that within each burst, the ISI-1 distributions of T305D mice as a population are both visibly and quantitatively more variable than those of WT control littermates. We found similar results for ISI-2, and ISI-3 (not shown). We did not use the usual measures like Standard Deviation (SD) or Coefficient of Variation (CV) for this analysis, since, as seen in Figure 4, the ISI histograms are truncated at 15 ms; the definition of the burst tends to underestimate the SD and CV for the T305 group. Nevertheless, SD was higher in the T305D group, but this difference did not reach statistical significance (3.19 for T305D vs. 3.10 for WT, p = 0.19).

#### Decreased reliability of bursts as position indicators

Since bursts are fewer in number compared to the overall number of spikes, and each session lasted only 30 minutes, the data collected were insufficient to perform a rigorous spatial regression fit to predict bursting given spatial position, as it was done for individual spikes earlier. Instead, we asked how reliably a burst reflected the animal's position in the place field. For example, if 100% of the bursts occurred when the animal is within the place field, the bursts would be deemed completely reliable. On the other hand, if bursts were randomly distributed outside place fields, one may conclude that bursting provides no added information about the animal's location. Since the overall place field sizes were different for T305D and WT mice, we defined the place field as those pixels with higher than average firing rate. We found that in T305D mice a significantly *lower* fraction of total bursts was contained in the PF compared to WT (71.9% for T305D vs. 77.4% for WT, p<0.05). This leads us to conclude that *bursts* are more variable and thus are a less reliable signal of position in T305D mice. We also repeated the same analysis by redefining the place field as the top 25% pixels by firing rate. Here also, we found that a lower fraction of bursts occurred within the place field for T305D (63.1% for T305D vs. 72.2% for WT, p<0.01). We also compared the spatial coherence of the *burst place-map* between the two groups. T305D mice had significantly lower spatial coherence (0.28 for T305D vs. 0.34 for WT, p<0.05). In other words, bursting intensity of nearby locations was not as correlated for T305D as for WT mice. This observation is in agreement with the spike place-field spatial coherence we noted earlier.

## Discussion

Previous studies demonstrated that α-CaMKII has a role in the stability of hippocampal place fields: Transgenic mice that express a mutated Ca^2+^-independent form of α-CaMKII show place cells that are both less precise and less stable [30]. Similarly, studies of mice with a mutation that substituted threonine 286 for alanine (α-CaMKII T286A) also revealed unstable place cells [23]. Both of these α-CaMKII mutations impaired hippocampal CA1 N-Methyl-D-Aspartate Receptor (NMDAR) dependent LTP as well as hippocampal-dependent learning (e.g. spatial learning)[30], [31]. These and other results indicated that hippocampal CA1 LTP is required for the stability of place cells [32], [33]. Here, we describe *in vivo* electrophysiological studies suggesting that besides a role in the stability of place fields, α-CaMKII is also implicated in shaping bursting patterns.

Besides unstable place fields with lower spatial coherence, α-CaMKII T305D mutant mice show dramatic changes in both the *intra*-burst and *inter*-burst properties of hippocampal place cells. Comparisons between T305D and WT groups showed that although the frequency of bursts did not differ significantly between the two groups, burst length (number of spikes per burst), average inter-burst intervals, and average intra burst intervals were altered in the mutants.

In addition, both inter-burst and intra-burst intervals were more variable in place cells of T305D mice, demonstrating that this mutation introduced high variability in the temporal structure of spike patterns. Spatial selectivity (elevation in firing rate within the place field) appeared to be lower in the T305D mutants, but this did not reach statistical significance. The variability in spike patterns of T305D mutants may have also affected other properties of spike bursts, perhaps accounting for the decreased spatial coherence and larger place fields of T305D mice. Thus, it is possible that the greater variability of bursting patterns of T305D mice contributed to their spatial learning deficits [1].

What could be the mechanism responsible for the changes of bursting patterns in T305D mice? Electrophysiological studies in brain slices indicated that this kinase modulates intrinsic excitability by regulating various ion currents. CaMKII may phosphorylate and regulate T-type Ca^2+^ channels thought to modulate the initiation of dendritic and somatic Ca^2+^ spikes involved in shaping spike patterns [34]. There is also a significant amount of evidence that implicates CaMKII in the modulation of A currents. CaMKII phosphorylates synapse dependent protein 97 (SAP97), and this phosphorylation regulates the post-synaptic density and dendritic localization of a key constituent of A currents (Kv4.2) [3], [35], [36]. A-type potassium currents (and Kv4.2) were implicated in the regulation of dendritic excitability and plasticity [36]. These findings are consistent with results from Drosophila showing that CaMKII inhibition with KN-62 or KN-93 caused a significantly decreased A-type current and resulted in abnormal firing patterns, including increased variability in spike frequency, inter-spike-interval, spike duration and amplitude [5]. Hippocampal neurons from α-CaMKII null mutants and rat neurons treated with a CaMKII inhibitor showed increased neuronal excitability and preponderance for both spontaneous and evoked seizures [37]. Cultured hippocampal neurons treated with a CaMKII inhibitor showed abnormal spike rates [38].

CaMKII is also thought to modulate the slow component of post-burst afterhyperpolarization (sAHP), a current known to shape spike patterns, since α-CaMKII T286A mutant mice showed a decrease in hippocampal sAHP following tetanic synaptic stimulation[39]. CaMKII may also modulate (directly or indirectly) the slowly activating h current, a key regulatory component of neuronal firing. Postsynaptic theta-burst firing can decrease neuronal excitability in a h-channel dependent manner. This decrease in excitability is also CaMKII-dependent, since an inhibitor of this kinase prevents it [6]. CaMKII-mediated phosphorylation of high-conductance, Ca^2+^-activated and voltage-gated (BK) channels is known to increase channel activity, and these channels have a key role in neuronal firing [40]. Additionally, there is also evidence that CaMKII modulates the expression and localization of G-protein-gated inwardly rectifying potassium (GIRK) channels [41]. Thus, CaMKII regulates a number of currents that are known to affect neuronal excitability and modulate spike patterns. However, prior to the present study there was little *in vivo* evidence demonstrating that this kinase had a role in shaping spike patterns.

Our results provide direct *in vivo* evidence that besides a role in the stability of hippocampal place fields (likely due to its involvement in the induction of LTP), α-CaMKII also modulates the temporal structure of spike patterns. Thus, the results presented here suggest that some of the molecular processes involved in acquiring information may also shape the patterns used to encode this information.

## Materials and Methods

### Ethics Statement

All procedures used in this work were reviewed and approved by the Chancellor's Animal Research Committee (ARC) at the University of California at Los Angeles, in accordance with US National Institutes of Health guidelines (ARC Protocol #: 1998-069).

### Subjects and Experimental Setups

The generation of the mutant mice used in the present study is described in detail elsewhere [1]. Place cells were recorded from 6 male mutant mice (C57/BL6J X 129SvJ genetic background, henceforth referred to as T305D mice) and 6 male littermate wild-type mice (henceforth WT) while the animals were freely moving inside a gray cylindrical chamber of 30 cm diameter with a height of 35 cm. Animals were food deprived to maintain 85∼90% of their original weight and trained to forage for food pellets. During the duration of the experiment, animals had unrestricted access to water. The slight food deprivation motivated mice to explore every corner of the recording chamber for all 3 recording sessions.

### Surgery

For the surgery, animals were anesthetized with Nembutal (50 mg/kg, i.p.) and treated with atropine methyl nitrate (0.4 mg/kg) for electrode implantation. Using a stereotaxic instrument, one tetrode mounted on movable microdrive was chronically implanted just above the CA1 pyramidal layer of the hippocampus (right hemisphere; AP −1.94, ML +1.5, DV −1.0). To position the electrode, the skull of the animal was exposed, and small holes were made over the target area for electrode bundle insertion. Five small screws were secured to the skull to help anchor the electrode assembly using dental acrylic mixture. One of the screws was extended with a connector to be used as a ground wire during recording. During the surgery, ophthalmic ointment was applied to the eye balls of the animal to prevent dryness. After 7 days of postoperative recovery, recordings were performed every day over several days while animals foraged for food pellets.

Tetrodes and stereotrodes were constructed using four 12.5 micron nichrome wires (Form Var coated, H.P. Reid, USA) each; an additional micro-wire was used for the ground. The tip of electrodes was cut with a 45° angle to yield maximum conductive area while the rest of the wires were insulated along their main axes. Each wire tip was gold-plated (Sifco Process, Independence, OH, USA) to obtain a final impedance of 300–500 kΩ (tested at 1 kHz).

### Screening and Recordings

Both screenings and recordings were performed in an isolated compartment constructed by the investigators. Inside the compartment, soundproof insulators were installed to avoid distraction of the animals by unwanted noise. A cylindrical black curtain was installed from ceiling to bottom, which provided a homogeneous visual environment around the recording cylinder. Recording cables were connected to an 8-channel amplifier (Neural-Lynx Co., Bozmen, MT, USA.) and then to the computer. At the end of the cable, a house-made 4-channel head-stage amplifier was installed. On the inside of the cylindrical chamber, a white cardboard (90° of arc) was installed as a local cue. Our set up also included a light positioned outside of the arena in the ceiling of the recording environment, which was a salient distal cue within the relative darkness of the recording environment.

On top of the compartment, a food pellet dispenser (Med-associates. Inc., St. Albans, VT, USA) was programmed to drop 20 mg food pellets into the recording chamber at random locations.

In increments of approximately 8–15 µm (up to a total of 25 µm daily), the electrodes were lowered gradually into the pyramidal cell layer of the CA1 region to isolate single neuron(s). The electrical signal from neurons, measured with respect to the ground, transmitted through the head stage amplifier (unity Gain buffer), through the cable to the programmable amplifier (gain between 5,000 to 20,000; filter 300 Hz high pass and 10 KHz low pass; Neuralynx Co., Bozmen, MT, USA). The amplified signal was then digitized and stored on a personal computer. The Discovery software package (DataWave Tech, USA) was used to process the incoming signals. Each spike waveform thus isolated was digitized and stored for cluster analysis. Also, neuronal firing rates were compared to exclude those inconsistent with pyramidal cell firing rates. Two infrared LEDs mounted on the head stage were used to monitor the position of the animal simultaneously with the neural recordings. The positions of LEDs were captured by an overhead CCD camera (60 Hz refreshing rate) and these signals were translated into 2D coordinate values by the video tracking system. These position values were then stored in the personal computer along with their time stamps. Samples were obtained from approximately 700 square pixels of 1×1 cm inside the recording chamber. Both spike and positional events were synchronously time stamped and used to analyze the spatial firing characteristics of the cell.

Isolated place cells were recorded in 3 successive 25–30-min sessions. Each session was separated by a 3–4 minute break for untwisting the cable, wiping the floor of the apparatus with alcohol and for the 90° cue rotation. Place maps showing average cell firing rates at positions in the cylinder were constructed for individual sessions. At the end of each session, the animal was returned back to its cage, the recording chamber was cleaned, and the recording cables were untwisted. Also, between the first and the second sessions, the visual cue was rotated by 90° in a counter clockwise direction. At the end of the second session the cue was placed back in its original position by rotating it 90° clockwise, thereby making the sensory cues identical to those of session 1. Mice were repeatedly exposed to the same recording chamber over days until the end of the experiment. In case the cell being recorded drifted or was lost between sessions, that cell was excluded from the analyses.

### Data Analyses

Only well-isolated single units confirmed to be in the CA1 region by histology were used in the analyses. Data obtained via data acquisition software were cluster-cut into single units. Each cluster-cut unit was confirmed to be a signal from a single neuron by verifying that no spike counts existed under the first 1 ms in the inter-spike interval histogram within a single unit.

For place field analysis, Linux based R- program was used. The midpoint of two LEDs was calculated for each position sample, and used as the position of the animal's head. For each pixel, the total number of spikes was divided by the total time spent by an animal in the pixel. Firing rates per pixel over the whole session were used to construct a color map representing a place field of each recording session.

A stability index was obtained by calculating pixel-by-pixel correlation between place maps derived from two recording sessions chosen pair-wise. Another index for stability was deemed as the amount of rotation of the place maps between 2 sessions that yielded the maximum value of pixel-by-pixel cross-correlation. For finding the maximum cross-correlation, pixel-by-pixel correlations were performed with a place map successively rotated in 1° steps. The size of the place field was defined as the number of pixels that had firing rates above the overall cell firing rate for the session. All correlation values were transformed into Fisher's Z scores for parametric comparisons. Definitions of spatial information, coherence, spatial selectivity, and variability, of complex spike bursts are defined in the corresponding sections of Results. The firing properties were calculated using data from all 3 sessions combined. Throughout the analyses, a two tailed unequal variance Student's t-test for equality of means was used to compare groups.

### Histology

After completion of recordings, recording locations were verified. Mice were overdosed with Nembutal, an electrolytic lesion was made by passing current through the recording electrode (5–20 µA, 10 s), and then perfused transcardially with 3.7% formalin (1∶10 dilution of 37% formalin solution in 0.9% saline). Brains were extracted and further fixed in 3.7% formalin (1∶10 dilution of 37% formalin solution in ddH_2_O) for a week at room temperature. Coronal sections (50 µm) were cut through the entire hippocampus with a microtome cryostat. The sections were stained with cresyl violet and examined under a light microscope to determine recording sites.
